# Development of voice perception is dissociated across gender cues in school-age children

**DOI:** 10.1038/s41598-020-61732-6

**Published:** 2020-03-19

**Authors:** Leanne Nagels, Etienne Gaudrain, Deborah Vickers, Petra Hendriks, Deniz Başkent

**Affiliations:** 1 Center for Language and Cognition Groningen (CLCG), University of Groningen, Groningen, The Netherlands; 2 Department of Otorhinolaryngology/Head and Neck Surgery, University Medical Center Groningen, University of Groningen, Groningen, The Netherlands; 3 Research School of Behavioral and Cognitive Neuroscience, Graduate School of Medical Sciences, University of Groningen, Groningen, The Netherlands; 4 CNRS UMR 5292, Lyon Neuroscience Research Center, Auditory Cognition and Psychoacoustics, Université de Lyon, Lyon, France; 5 Cambridge Hearing Group, Clinical Neurosciences Department, University of Cambridge, Cambridge, United Kingdom

**Keywords:** Language, Perception

## Abstract

Children’s ability to distinguish speakers’ voices continues to develop throughout childhood, yet it remains unclear how children’s sensitivity to voice cues, such as differences in speakers’ gender, develops over time. This so-called voice gender is primarily characterized by speakers’ mean fundamental frequency (F0), related to glottal pulse rate, and vocal-tract length (VTL), related to speakers’ size. Here we show that children’s acquisition of adult-like performance for discrimination, a lower-order perceptual task, and categorization, a higher-order cognitive task, differs across voice gender cues. Children’s discrimination was adult-like around the age of 8 for VTL but still differed from adults at the age of 12 for F0. Children’s perceptual weight attributed to F0 for gender categorization was adult-like around the age of 6 but around the age of 10 for VTL. Children’s discrimination and weighting of F0 and VTL were only correlated for 4- to 6-year-olds. Hence, children’s development of discrimination and weighting of voice gender cues are dissociated, i.e., adult-like performance for F0 and VTL is acquired at different rates and does not seem to be closely related. The different developmental patterns for auditory discrimination and categorization highlight the complexity of the relationship between perceptual and cognitive mechanisms of voice perception.

## Introduction

Voice cues enable listeners to recognize and distinguish speakers, which is imperative for speech-related tasks, such as speech perception in noisy environments. At an early age, children are already sensitive to differences in voice cues, especially for familiar speakers^1^ and in their native language^2^. Furthermore, infants are already sensitive to differences in voice cues, such as fundamental frequency (F0)^3^ or voice pitch^4^, timbre differences associated with vocal-tract length^5^, or prosody^6^. On the other hand, there is a clear prolonged development in children’s ability to encode and recognize the characteristics of voices^7–9^. Hence, it is unclear how children’s sensitivity to differences in voice cues develops over time.

Children’s ability to discriminate differences in voice-related acoustic cues, such as F0^10–13^ or temporal cues^14,15^, continues to develop throughout childhood. Yet, the specific age at which children’s voice discrimination thresholds are adult-like has been debated, partially due to differences in experimental stimuli. Earlier research has primarily used non-voice stimuli, such as pure tones^10,11^, or octave-band^14^ and narrow-band noises^15^, where the results were interpreted and extrapolated for voice perception. In contrast, research directly using voice stimuli has been scarce^12,13^. Further challenges come from task demands, as these can influence outcomes, and the high variability among children’s performance, even after controlling for effects of age^16^. Children’s ability to categorize voice and speech cues, a higher-order cognitive task, also continues to develop even after 12 years of age, e.g., as observed for phonemic categorization^17,18^. These higher-order cognitive identification tasks depend on long-term exposure to voice cues and stored representations of voice categories in addition to perceptual processing^19,20^. Despite these shared features, the source of the relatively slow development in sensitivity to differences in voice cues and the relation between discrimination and categorization of voice cues are not well understood. Some parts of the auditory system only become mature during or after childhood, such as the auditory cortex, as evidenced by developmental effects on the latency of evoked cortical potentials^21^, but children’s sensitivity to subtle acoustic cues seems to be also largely determined by cognitive development and auditory experience^22,23^.

Mann, Diamond, and Carey^7^ have proposed that children’s difficulties with encoding speakers’ voice characteristics are caused by changes in central auditory processing related to right hemisphere maturation. This explanation is partially supported by findings of recent neuroimaging studies which show that listeners rely on areas in both hemispheres to process differences in speakers’ voices, but predominantly on the right hemisphere^24,25^. Furthermore, Mann *et al*.^7^ proposed that the development in children’s ability to encode unfamiliar speakers’ voice characteristics may be caused by different processing strategies and reliance on different acoustic cues compared to adults. This hypothesis is in agreement with the ‘Developmental Weighting Shift (DWS)’ model for categorical perception of phonemes by Nittrouer and Miller^18^, which states that children weigh dynamic acoustic cues of speech (such as formant transitions) more than static acoustic speech cues (such as noise segments of consonants) due to their higher perceptual salience and more informative properties. The usage of static acoustic cues seems to require more auditory experience and language exposure for children to attend to these cues. A similar explanation may apply to the development of children’s sensitivity to different acoustic voice cues, such as voice gender cues.

The perceived gender of a voice results from a combination of many acoustic features^26,27^. However, it is primarily defined by the mean F0, related to glottal pulse rate, and vocal-tract length (VTL), related to the size of the speaker^28^. The mean F0 of female voices is around 70%, approximately 12 semitones (st), equal to one octave, higher than male voices^29^, but, as F0 can be modulated easily within the same speaker, the range of F0 values of male and female voices largely overlap. Therefore, listeners are exposed to a wide range of F0 variations. F0 contours indicate a word’s meaning in tonal languages and play a large role in prosodic differences that convey, among others, the emotions and attitudes of speakers, and they are an important cue for infants to recognize their mother’s voice^4^. Therefore, F0, with its information-bearing dynamic fluctuations, seems to parallel dynamic formant transitions in the DWS model and may consequently be subject to similar principles.

The female vocal-tract is around 20% shorter than the male vocal-tract, causing a difference of around 3.6 st in formant frequencies^26^. Estimation of speakers’ VTL is essential for listeners to recognize speech, as the formant frequency distributions of vowels depend on speakers’ VTL size^30^. Differences between speakers’ mean F0 and VTL both facilitate speech perception in competing speech maskers for adult listeners^31,32^. VTL cues are unlike F0 cues in that they can only be modified to a very limited extent within a speaker. This reduces the range of different values and fluctuations that listeners are exposed to relative to F0, behaving more like the static cues of the DWS model. Previous research on voice gender categorization in adult listeners has also indicated that differences in dynamic F0 cues may be more perceptually salient^33,34^ than static VTL cues. Finally, there are differences in the mean F0 and VTL values of male and female voices across speakers of different languages and modalities. For an overview of mean F0 values for different languages and modalities see^35^, and for an overview of differences in average height per country, which is the main determinant of speakers’ VTL^28^, see^36^.

For children’s voices, voice gender is differentiated almost exclusively by formant frequencies, as there is a large overlap in mean F0 values until around the age of 12^37,38^. For instance, Perry *et al*.^37^ found that the formant frequencies of 8-year-old boys were approximately 9% lower than those of 8-year-old girls, while their F0 values did not significantly differ from each other. As there are also no gender differences in the VTL size of children before 12 years of age, Vorperian and Kent^38^ have proposed that the gender differences in children’s formant frequencies may arise from differences in the resonator width and size instead of the length of the vocal-tract. For face recognition, Anastasi and Rhodes^39^ found that children and adults are better at recognizing faces from people of their own age. This ‘own-age bias’ may also apply to children’s ability to recognize voices. Consequently, children may attend to different acoustic cues than adults, and hence have different representations of voice gender categories than adults.

In the present study, we investigated whether children’s ability to discriminate voice gender cues (Experiment 1) and how they weigh voice gender cues for categorization (Experiment 2) develop similarly for F0 as for VTL cues, the primary cues of voice gender, and at what age children’s performance is adult-like. Previous studies have shown that children’s discrimination of F0 continues to develop throughout childhood, but some of these studies measured pure-tone frequency discrimination using non-voice stimuli^11,40^ instead of discrimination of more realistic voice pitch cues using voice stimuli^12,13^. Regarding VTL, discrimination has only been studied before in infants and only using EEG, which, while providing some evidence of processing of the acoustic traits associated with VTL, does not necessarily reflect perception^5^. Thus, the development of children’s ability to discriminate differences in voice gender cues has not been previously studied in a systematic way. Furthermore, the relation between children’s discrimination, a lower-order perceptual ability, and the categorization of voice cues, a higher-order cognitive process, is not well understood. If children’s ability to discriminate F0 and VTL cues is indeed reduced, their weighting may differ from adults’ due to perceptual limitations. However, as discrimination is a lower-level perceptual task and categorization is a higher-level cognitive task, the development of both abilities may also be dissociated. We conducted two experiments to measure children’s just noticeable differences (JNDs), i.e., discrimination thresholds, for F0 and VTL (Experiment 1), and their perceptual weighting of F0 and VTL cues for the categorization of voice gender (Experiment 2). We hypothesized that children are more sensitive to differences in F0 than VTL, as F0 is a more dynamic cue that varies widely among and within speakers, and therefore listeners are exposed to wide variations in F0. These characteristics may make F0 more perceptually salient than VTL, which can be modified only to a very limited extent within the same speaker, and hence lead to a higher weighting of F0, similar to principles of the DWS model. Alternatively, due to high overlap in speakers’ F0 values across gender, VTL may be a more informative cue for determining speakers’ voice gender, and hence receive a higher weighting than F0. In addition, we expected children to show different weighting of F0 and VTL cues for voice gender categorization than adults due to differences in their representations of voice gender categories, based on differences in exposure to voice cues and an ‘own-age bias’^39^. Finally, we investigated if children’s discrimination and weighting for voice gender categorization develop in a similar manner for F0 and VTL, as these abilities may rely on different auditory processes.

## Experiment 1 - Discrimination of F0 and VTL cues

### Methods

#### Participants

Fifty-eight children between the ages of 4 and 12, divided into four different age groups (Table 1), and fifteen adults between the ages of 20 and 30 took part in the study. All participants were native speakers of Dutch, and reported no history of hearing or language disorders. We screened participants’ hearing thresholds at 20 dB HL using pure-tone audiometry at octave frequencies between 500 and 4000 Hz to ensure they were normal hearing. Table 1 summarizes the participants’ demographic characteristics. Children’s vocabulary size was measured using the Dutch version of the Renfrew Word Finding Vocabulary Test^41^ to ensure age-normal linguistic development in our test population.

**Table 1:** Demographic characteristics of participant age groups.

Participant age groups	Number of participants	Age (median)	Gender (f:m)	Vocabulary (median)
Children 4–6 years	13	5.08	8:5	36
Children 6–8 years	13	7.17	7:6	40
Children 8–10 years	16	9.00	10:6	45
Children 10–12 years	16	11.10	10:6	46
Adults	15	24.40	11:4	—

Age is given in decimal years. Vocabulary corresponds to scores on the Renfrew Word Finding Vocabulary Test (maximum score of 50 points).

A written informed consent form for study participation was signed by the parents and/or legal guardians of children and by adult participants prior to data collection. Ethical approval of the study was given by the Medical Ethical Review Committee of the University Medical Center Groningen (METc 2016.689). All experiments and methods were performed in accordance with the relevant guidelines and regulations.

#### Stimuli and apparatus

The stimuli consisted of three-syllable CVCVCV nonwords, for instance, ‘ba-ki-mo’, that were spliced from Dutch CVC words, taken from the NVA corpus^42^. The stimuli were produced by a female native speaker of Dutch with a mean F0 of 242 Hz and an estimated VTL size of approximately 13.5 cm, based on the average height of Dutch women of 168.72 cm^36^. For the experiment, we focused on the differences in F0 and VTL relative to the original speaker’s voice. As both F0 and VTL alterations lead to differences in frequencies, we expressed the differences as ratios measured on a logarithmic scale in st instead of differences in Hertz or centimeters. The word recordings were spliced into 61 CV syllables, equalized for root-mean-square (RMS) level, and analyzed using STRAIGHT^43^, to obtain the F0 contour and the spectral envelope. The method that we used is the same as employed by Gaudrain and Başkent^44^.

For each trial, three CV syllable pairs were randomly selected, normalized to a duration of 200 ms and a mean F0 of 242 Hz, and resynthesized with STRAIGHT using the new F0 and VTL parameters. This resynthesis procedure was performed for all stimuli to prevent potential artifacts arising from the resynthesis procedure itself. The three syllables were then concatenated with 50 ms of silence separating the individual syllables. The overall F0 contour of a CVCVCV nonword was adjusted by shifting the natural F0 contour of each CV syllable by random steps of 1/3 st, which could be −1/3 st, 0 st or +1/3 st, to make them sound more natural. After adjusting the F0 contour of each CV syllable, the overall F0 contour of the nonword was centered to zero again so it would not affect the mean F0. For the F0 manipulation, the F0 contour (in Hz) was multiplied by a specified factor, which depended on the applied change in mean F0, to preserve the original F0 fluctuations and only adjust the mean F0. For the VTL manipulation, the spectral envelope was compressed toward the lower frequencies to generate an overall spectral shift. The modified F0 contour and spectral envelope were then recombined using a pitch synchronous overlap-add (PSOLA) resynthesis method.

In each trial, the same nonword with the same syllable structure was used for the target nonword and the two standard nonwords. The target nonword differed in mean F0 or VTL from the two standard nonwords. Besides this manipulation, there were only small differences in the mean F0 contour of the nonwords to make them sound more natural. Depending on the condition, the target nonword was either produced with a lower F0 or a larger VTL value relative to the original female speaker’s voice, which made the target nonword sound more masculine. All signal processing was performed in Matlab^45^ using a sampling frequency of 44.1 kHz.

#### Procedure

We used a 3-interval 3-alternative forced-choice (3I-3AFC) adaptive procedure to measure participants’ JNDs for F0 and VTL. The experiment started with two training sessions, each consisting of three trials, and was followed by two experiment sessions of approximately 25 to 45 trials, depending on participants’ responses. The experiment sessions were presented to participants in a randomized order, either measuring their F0 or VTL JND first.

Each experiment session started with a difference of 12 st in F0 or VTL values relative to the original female speaker’s voice. The voice difference decreased by one step size after two consecutive correct responses and increased by one step size after an incorrect response (2-down, 1-up). The initial step size was 2 st but after 15 trials with the same step size or when the difference became smaller than two times the step size, the step size was divided by √2. The experiment session ended after eight reversals. Finally, we calculated the geometric mean of the voice difference values at the last six reversals to determine participants’ JND, which corresponds to the 70.7% correct discrimination point on the psychometric function^46^.

**Figure 1:**
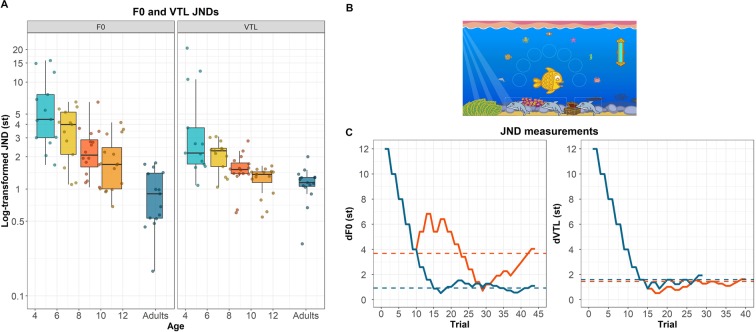
Differences in JNDs for F0 and VTL, the experimental interface of Experiment 1, and the adaptive tracks of one adults and one child participant. (**A**) Log-transformed JNDs for F0 (left) and VTL (right). The dots show individual data points located at participants’ age in years, rounded to two decimal places (N_children_ = 58; N_adults_ = 15). The boxplots depict the median per age group, and the box shows the lower and upper quartiles. The whiskers show the lowest and highest data points within plus or minus 1.5 times the interquartile range. (**B**) The experimental interface. As participants give correct answers, they collect sea animals around the central yellow fish. The illustrations were made by Jop Luberti for the purpose of this study. This image is published under the CC BY NC 4.0 license (https://creativecommons.org/licenses/by-nc/4.0/). (**C**) The F0 and VTL JND adaptive tracks of one adult (NHA012) and one 8- to 10-year-old child participant (NHK034).

Stimuli were presented to participants on a touchscreen laptop via Sennheiser HD 380 Pro headphones at a sound level of 65 dBA. A child-friendly interface was constructed using Matlab to maintain children’s attention (Fig. 1B). In each trial, three sea animals appeared on the screen and subsequently produced the same CVCVCV nonword. Participants were instructed to click on the sea animal whose voice differed from the other two sea animals. Visual feedback on response accuracy was provided to participants. Children were tested in a quiet room at their homes, and adults were tested in a quiet testing room at the University of Groningen.

#### Data analysis

Participants’ JNDs for F0 and VTL were analyzed using the lme4 package (version 1.1.12^47^) in R (version 3.4.1^48^). A linear mixed-effects model with a two-way interaction between *voice cue* (F0 or VTL) and *age*, and a random intercept per participant for children’s data was computed to examine if children’s discrimination of F0 and VTL cues developed similarly. We evaluated the models using backward stepwise selection with ANOVA Chi-Square tests, starting with the full factorial model, in lme4 syntax: JND log ~ voice cue*age + (1|participant). We used log-transformed JNDs, which are more normally distributed than the raw values, as the JND procedure does not allow crossing the zero line. Furthermore, the log-transformation reduced the differences in variability and decoupled it from the mean. *Voice cue* indicated whether the JND was for F0 or VTL, while *age* represented children’s age in decimal years. In addition, to investigate at what age children’s discrimination of F0 and VTL does not differ from adults, we used the Desctools package (version 0.99.25^49^) to perform two Dunnett’s Tests on subsets of F0 and VTL JNDs with *JND log* as an outcome variable and *age group* as a fixed effect.

## Results

### Do children’s discrimination abilities develop similarly for F0 and VTL as a function of age?

Fig. 1A shows participants’ JND values for F0 (left) and VTL (right) as a function of their age (dots) and the median JND per age group (boxplots), Fig. 1B shows the experimental interface, and Fig. 1C shows the F0 and VTL JND adaptive tracks of one adult and one child participant. We first performed a Shapiro-Wilk test to confirm that the assumption for LMMs of normally distributed residuals for our data was met (*W* = 0.98, *P* = 0.13). Model selection revealed that the model with *voice cue* and *age* as fixed effects had a better and more parsimonious fit than the model with the two-way interaction between *voice cue and age* [χ^2^(1) = 0.02, *P* = 0.89], or the models without *voice cue* [χ^2^(1) = 24.80, *P* < 0.001] or without *age* [χ^2^(1) = 28.01, *P* < 0.001] as fixed effects. The significant effects of *voice cue* and *age* indicate that children’s JNDs were significantly lower for VTL than for F0, [*F*(1,56) = 30.95, t value = −5.56, *P* < 0.001], and generally decreased significantly with age [*F*(1,56) = 36.01, t value = −6.00, *P* < 0.001]. However, the rate of improvement, i.e., the slope size, did not significantly differ across cues, which is evidenced by the lack of a significant interaction between *voice cue* and *age*.

### At what age do children show adult-like discrimination of F0 and VTL?

We performed two Dunnett’s Tests on subsets of F0 and VTL JNDs per age group. For F0, the JNDs of adults significantly differed from the JNDs of children of all age groups [4–6 years: diff = 1.85, *P* < 0.001; 6–8 years: diff = 1.38, *P* < 0.001; 8–10 years: diff = 1.01, *P* < 0.001; 10–12 years: diff = 0.75, *P* < 0.01]. For VTL, adults’ JNDs significantly differed from the JNDs of 4- to 8-year-olds [4–6 years: diff = 1.07, *P* < 0.001; 6–8 years: diff = 0.62, *P* < 0.01] but not from the JNDs of 8- to 12-year-olds [8–10 years: diff = 0.30, *P* = 0.30; 10–12 years: diff = 0.09, *P* = 0.96].

## Discussion

Our results show that children’s discrimination of F0 and VTL improves gradually with age, but discrimination of VTL becomes adult-like earlier than their discrimination of F0. Adults’ VTL JNDs did not significantly differ from those of 8- to 12-year-old children, whereas adults’ F0 JNDs differed from children’s F0 JNDs at all ages. The increase in sensitivity to differences in F0 cues with age is in line with previously reported research^10–13^. The differences in the reported ages at which children’s discrimination of F0 is adult-like are likely due to differences in stimuli and task demands. The perception of VTL differences has only been studied before in infants and adults, using an objective EEG mismatched negativity paradigm^5^. Vestergaard *et al*.^5^ found that newborn infants are already sensitive to large differences in VTL, but it was not possible to quantify infants’ ability to discriminate VTL and to assess whether it was adult-like yet. To our knowledge, there is no prior information regarding the development of school-age children’s discrimination of VTL differences.

Our findings do not support our expectation that children would be more sensitive to differences in F0 than VTL due to exposure to wide variations in F0, even within the same speaker, compared to small variations in VTL, which usually only differs markedly between different speakers. According to the DWS model^18^, children are more sensitive to the most salient and informative acoustic cue, which can be F0 because of its higher perceptual salience^33,34^ or VTL because of its lower overlap across genders and potential higher informativeness for determining speakers’ voice gender. Also on a linguistic level, VTL seems to be more informative, as it is required for the correct interpretation of vowels^30^, while F0 primarily provides paralinguistic information in non-tonal languages. However, the DWS model is also based on categorization rather than discrimination of acoustic cues. Acoustic dynamics may have different effects on discrimination than on categorization. Evidence for a potential trade-off effect between the discrimination and categorization of acoustic cues based on acoustic dynamics is provided by Mirman, Holt, and McClelland^50^. They found that adult listeners could more accurately discriminate sounds that differed in steady-state acoustic cues than rapidly-changing, dynamic acoustic cues, but they showed an opposite benefit for categorization of these cues. Contrary to our expectation, dynamic cues may be more difficult to discriminate than static cues but perhaps still more perceptually salient for categorization.

Another explanation is that children may have different representations of voice gender than adults, as discrimination abilities are also influenced by the categories that listeners have of the particular acoustic dimensions^51,52^. Voice gender differences in children’s voices are primarily caused by differences in formant frequencies, possibly due to differences in the resonator width and size^38^, as F0 and VTL values do not systematically vary with sex until puberty^37,38^. As a result, children may have voice gender representations that are primarily based on voice gender differences among children’s voices instead of adults’ voices.

## Experiment 2: F0 and VTL weighting for voice gender categorization

### Methods

#### Participants

The same group of participants that took part in the first experiment also participated in the second experiment.

#### Stimuli and apparatus

The stimuli consisted of four CVC words, *bus* [bus], *vaak* [often], *leeg* [empty], and *pen* [pen], produced by the same female speaker with a mean F0 of 201 Hz and taken from the same corpus as used for Experiment 1^42^. The recordings were first equalized in RMS and modified using STRAIGHT^43^. The F0 and VTL values were manipulated independently from each other in the same manner as the stimuli of Experiment 1. For the condition in which stimuli had differences of 0.0 st in F0 and 0.0 st in VTL, stimuli were also resynthesized using the same procedure to prevent any artifacts from the resynthesis procedure itself.

The F0 was decreased by 0.0 st, 6.0 st, or 12.0 st, resulting in mean F0 values of 201 Hz, 142 Hz, and 100 Hz respectively, while the VTL was increased by 0.0 st, 1.8 st, or 3.6 st, corresponding to the estimated VTL sizes of 13.5 cm, 15.1 cm, and 16.6 cm. Thus, there were nine different voice combinations for the four selected words, resulting in 36 stimuli. The values for the differences in F0 and VTL were chosen based on earlier studies on gender categorization by Smith and Patterson^53^ and Smith *et al*.^54^, which in turn were derived from the voice gender differences in the Peterson and Barney^55^ vowel database. Seven out of nine voice combinations that were used in our experiment were within 95% of the adult population based on these values. In addition, Fuller *et al*.^56^ showed that a combined difference of a decrease of 12 st in F0 and an increase of 3.6 st in VTL using this manipulation procedure reliably changes the perceived gender of speakers for adult listeners. The number of “male” categorizations was less than 10% when only the F0 of the speaker was decreased by 12 st and around 30% when only the VTL of the speaker was increased by 3.6 st.

#### Procedure

A visual-auditory match-to-sample task was used to investigate children’s weighting of F0 and VTL cues for voice gender categorization. The experiment consisted of a training session of 5 trials and an experiment session of 36 trials with items that were presented in a randomized order. In each trial, a stimulus word was produced with voice characteristics in which the F0 and VTL parameters were more female, i.e., conforming more to the original speaker’s voice parameters, more masculine, because of a decrease in F0 and an increase in VTL, or somewhat ambiguous.

**Figure 2:**
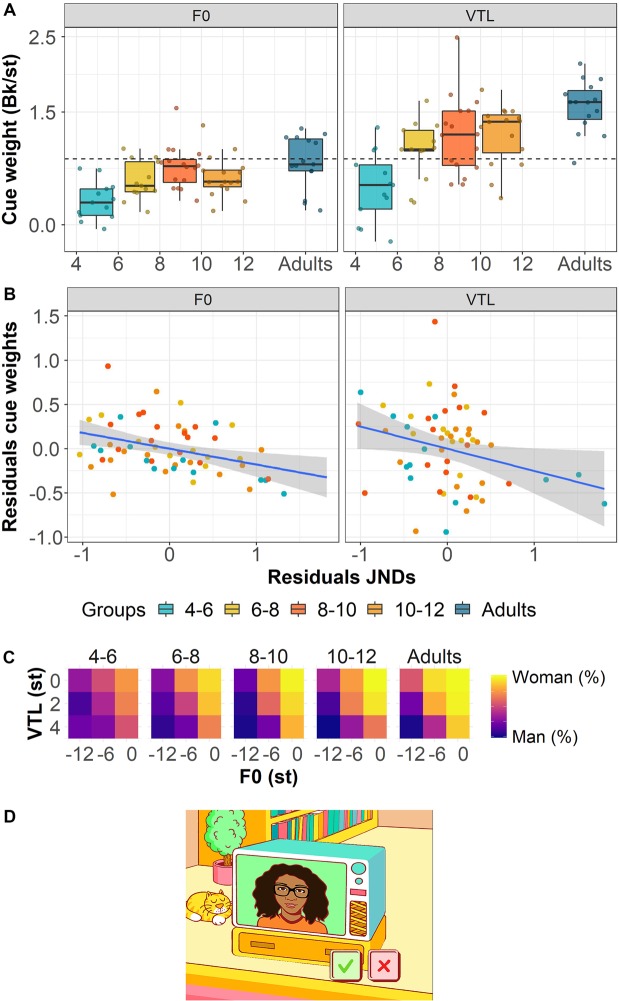
Cue weights for F0 and VTL on the voice gender categorization task, the correlations between participants’ JNDs and cue weights, and the experimental interface of Experiment 2. (**A**) Participants’ cue weights for F0 (left panel) and VTL (right panel). The dots show individual data points at participants’ age in years, rounded to two decimal places (N_children_ = 58; N_adults_ = 15) and the boxplots show the median cue weights per age group. The box shows the lower and upper quartiles, and the whiskers show the lowest and highest data points within plus or minus 1.5 times the interquartile range. (**B**) The correlations between participants’ F0 JND and cue weight residuals (left panel) and VTL JND and cue weight residuals. (**C**) Average gender categorization judgments as a function of differences in F0 (x-axis) and VTL (y-axis) in st for each individual age group. Blue corresponds to 100% “man” categorizations and yellow corresponds to 100% “woman” responses. (**D**) The experimental interface. Participants were instructed to click on the green check mark if the perceived gender of the portrayed face and voice matched and on the red cross if they did not match. The illustrations were made by Jop Luberti for the purpose of this study. This image is published under the CC BY NC 4.0 license (https://creativecommons.org/licenses/by-nc/4.0/).

The stimuli were presented using the same touchscreen laptop and Sennheiser HD 380 pro headphones as were used in Experiment 1. The experiment was conducted using a child-friendly interface that was developed in Matlab (Fig. 2D). After hearing the auditory stimulus, participants were presented with either a male or female face on the computer screen. Participants could then indicate whether the voice and the face were of the same gender by pressing a green check mark or a red cross when the gender of the voice and face differed. No feedback on the accuracy of responses was provided to participants. The second experiment was conducted in the same testing environment after participants completed the first experiment.

#### Data analysis

We analyzed participants’ perceptual weighting of F0 and VTL cues for voice gender categorization across age. To calculate the *cue weights*, we first normalized the F0 and VTL differences relative to the original speaker’s voice in st and defined them as *δF0* = *−ΔF0*/12 – 0.5 and *δVTL* = *ΔVTL*/3.6 – 0.5. This normalization was done to make F0 and VTL functionally equivalent for model fitting, despite the differences in range and quality. With these normalized cues, the original female speaker’s voice had a *δF0* value of −0.5 and a *δVTL* value of −0.5, while the most male-sounding voice (difference of −12 st in F0 and +3.6 st in VTL) had a *δF0* of + 0.5 and a *δVTL* of +0.5. We then extracted the coefficients of participants by fitting a mixed-effects logistic regression model with random intercepts and slopes for *δF0* and *δVTL* per participant, in lme4 syntax: response ~ (δF0 + δVTL | participant). This model gives coefficient predicting values on a logit scale, i.e., log odds ratios based on the natural log, relative to the normalized *δF0* and *δVTL* ranges. We converted the participants’ coefficients for *δF0* and *δVTL* into “Berkson” (Bk) units^57^ per st by scaling the factors so they correspond to log_2_ odds ratios per st. This conversion makes the differences in cue weights easier to interpret, as an increase of 1 Bk per st is equal to doubling the percentage of male categorizations relative to that of female categorizations. Individual Bk units of children were then analyzed by fitting generalized linear mixed-effects models with random intercepts per participant. Models were compared using backward stepwise model selection with ANOVA Chi-Square tests, starting with the full factorial model with *cue weight* (Bk/st) as an outcome variable and a two-way interaction between *voice cue* (F0 or VTL) and *age*, in lme4 syntax: cue weight ~ voice cue*age + (1|participant).

In addition, we used two Dunnett’s Tests on age group subsets of F0 and VTL cue weights to investigate at what age children’s cue weights were adult-like. Finally, we determined two Pearson’s correlation coefficients relating children’s F0 and VTL JNDs to their F0 and VTL cue weights. To ensure that the correlation between children’s JNDs and cue weights for F0 and VTL was not caused by a general effect of age, we extracted the residuals from four linear models. This method partials out the effect of age group on the differences in JNDs and cue weights. As outcome variables, we used either log JNDs or cue weights in Bk units and only *age* as a fixed effect. These analyses were repeated for F0 and VTL. The residuals of each model were then used to calculate the correlation between JNDs and cue weights for each cue.

## Results

### Does children’s cue weighting for voice gender categorization develop similarly for F0 and VTL as a function of age?

Fig. 2A shows participants’ cue weights for F0 (left panel) and VTL (right panel) as a function of their age (dots) and age group (boxplots). Figure 2B shows the correlation between participants’ cue weight residuals for F0 (left panel) and VTL (right panel) as a function of their respective JND residuals (Experiment 1) and age group. Figure 2C shows the average categorization judgments as a function of differences in F0 and VTL for each individual age group. Model selection demonstrated that the full model with a two-way interaction between *voice cue* and *age* had a significantly better fit than the model with *voice cue* and *age* as fixed effects [χ^2^(1) = 16.4, *P* < 0.001]. The significant effect of *voice cue* shows that children’s weighting of VTL was overall higher than their weighting of F0 [*Estimate* = 0.20, *t* = 2.74, *P* < 0.01]. On average, the ratio between F0 and VTL perceptual weights differed by 2% between children and adults. Yet, children gave 71% of the weight given to F0 by adults and 63% of the weight given to VTL. Furthermore, the significant effect of age shows that children’s weighting of both F0 and VTL cues became more adult-like with age [*Estimate* = 0.02, *t* = 2.64, *P* < 0.01]. Finally, the significant interaction between *voice cue* and *age* [*Estimate* = 0.02, *t* = 4.29, *P* < 0.001] indicates that there was an overall larger increase in children’s weighting of VTL with age relative to their weighting of F0. However, since there was also a significant fixed effect of *voice cue*, which indicates that participants’ overall weighting of F0 differs from that of VTL (when expressed in Berkson per semitone), the interaction could simply stem from the same multiplicative effect of age being applied at different intercepts. In a signal-detection-theory model^58^, the ratio between cue weights will be constant if the responses are affected by a single source of internal noise which is common to both cues. We thus further explored this interaction by looking at the *relative* contribution of the two cues. For this we calculated, for each participant, the ratio between weighting of F0 and VTL. We found that, after removing data of four 4- to 6-year-old children who showed extremely singular weighting values, the ratios between participants’ weighting of F0 and VTL did not differ across age [*F*(1,52) = 0.65, *P* = 0.42], which suggests that the interaction between *voice cue* and *age* was likely caused by the differences in the overall weighting of F0 and VTL.

### When does children’s weighting of F0 and VTL cues for voice gender categorization become adult-like?

The Dunnett’s Test that was performed on the subset of F0 cue weights indicated that adults’ F0 cue weights only significantly differed from 4- to 6-year-old children’s cue weights [4–6 years: diff = −0.51, *P* < 0.001]. For VTL cue weights, the Dunnett’s Test indicated that adults’ cue weights significantly differed from 4- to 10-year-old children’s cue weights but not from 10- to 12-year-old children’s cue weights [4–6 years: diff = −1.08, *P* < 0.001; 6–8 years: diff = −0.59, *P* < 0.01; 8–10 years: diff = −0.39, *P* < 0.05; 10–12 years: diff = −0.37, *P* = 0.061].

### Is children’s discrimination of F0 and VTL related to their cue weighting of F0 and VTL for voice gender categorization?

Two Pearson’s correlations between children’s JNDs and cue weights showed moderate correlations between F0 JNDs and cue weights [Pearson’s *r* = −0.49, *P* < 0.001] and VTL JNDs and cue weights [Pearson’s *r* = −0.48, *P* < 0.001]. However, the correlation was only significant for the group of 4- to 6-year-old children for F0 [Pearson’s *r* = −0.63, *P* < 0.05] and for VTL [Pearson’s *r* = −0.59, *P* < 0.05]. Hence, the reported correlations mainly seem to be driven by the generally low performance of some of the youngest children on both tasks. For this reason, we performed two additional Pearson’s correlations using the residuals of children’s F0 and VTL JNDs (Experiment 1) and cue weights (Experiment 2) to prevent a correlation resulting from a general effect of age. Figure 2 shows the correlation between participants’ F0 JND and cue weight residuals and their VTL JNDs and cue weight residuals (Experiment 2). We found that there was still a significant moderate correlation between the residuals of participants’ JND and cue weight residuals for F0 [Pearson’s *r* = −0.37, *P* < 0.01] and a small correlation for VTL [Pearson’s *r* = −0.28, *P* < 0.05] after this correction for a general effect of age. In addition, we looked again at the correlations between JND residuals and cue weight residuals for F0 and VTL for individual age groups, and we found that, as for the non-residuals, there was only a significant correlation for the 4- to 6-year-olds for F0 [Pearson’s *r* = −0.64, *P* < 0.05] and for VTL [Pearson’s *r* = −0.61, *P* < 0.05] JNDs and cue weights even after taking a general effect of age into account.

## Discussion

Our results show that children’s cue weights for F0 only differed from adults’ cue weights for the youngest age group between 4 and 6 years of age, while children’s cue weights for VTL differed from adults’ cue weights for the age groups between 4 and 10 years of age. We also investigated if participants’ JNDs and cue weights for F0 and VTL were related to each other. After correcting for a general effect of age, we only found a significant moderate and a small overall correlation for between JNDs and cue weights for F0 and VTL, respectively, but our additional correlation analyses for individual age groups showed that only 4- to 6-year-old children’s F0 and VTL JNDs and cue weights were significantly correlated.

Contrary to our first experiment, the results of our second experiment are in line with our expectation that children’s weighting of F0 cues for voice gender categorization is adult-like earlier than their weighting of VTL cues. F0 is a more dynamic acoustic cue than VTL and may be a more perceptually salient cue for children to use for voice gender categorization^18,33,34^. Hence, children may learn earlier which F0 values correspond to male and female voices compared to VTL values, as they may attend more to F0 differences. In keeping with the findings of Mirman *et al*.^50^, the different developmental patterns for F0 and VTL provide evidence for a trade-off effect between the discrimination and categorization of acoustic cues based on their acoustic dynamics (steady-state, static acoustic cues versus rapidly-changing, dynamic acoustic cues). A potential explanation for this observation is related to the fact that, in NH adults, the VTL JNDs represent a larger proportion of the typical male-female difference (about 34%) than F0 JNDs (about 9%). As a result, VTL appears less robust to perceptual degradations than F0, as was observed in adult cochlear implant users^44,56^. In the present study, the larger VTL JNDs observed in the younger age groups, which were sometimes above the smallest step size of 1.8 st in VTL, may have hindered children’s access to the VTL cue in the gender categorization task. However, as participants’ JNDs corresponded to the 70.7% correct discrimination point on the psychometric function, participants may have still been able to perceive F0 and VTL differences that were below their discrimination thresholds that were measured in Experiment 1. Evidence for this possibility is provided by the significant increase in children’s VTL weighting for the 6- to 8-year-old age group compared to the 4- to 6-year-old age group (Fig. 2).

Children’s weighting was adult-like earlier for F0 than for VTL, but they did use both acoustic cues for categorizing speakers’ voice gender at all ages. This finding implies that children may have different representations of voice gender and their corresponding F0 and VTL values than adults. Most children were consistent in their categorizations of the clearly male- and female-sounding voices, but they were less consistent in categorizing the other, more ambiguous sounding, voices. A similar finding was reported by Hazan and Barrett^17^ who found that 6- to 12-year-old children’s categorization of phonemes, based on a range of phonemic contrasts, was not as consistent and clear as it was for adults. One could also consider that children’s representations of voice gender may differ from adults because their representations are predominantly based on voice gender differences between other children rather than adults^39^. As the voice gender of child voices cannot be recognized reliably based on differences in their F0 or VTL values until the age of 12^37,38^, this may also explain why they show different weighting of these cues than adults.

Furthermore, as mentioned by Hazan and Barrett^17^, children’s limited attention span seems to play merely a minor role in explaining their inconsistent responses, as most children categorized the clearly male- and female-sounding voices consistently, apart from some children in the youngest age group. Children may not have clear concepts of what a male or female voice sounds like yet for various reasons. For instance, they may interact primarily with other young children for whom the differences in their perceived voice gender are still very limited^37,38^. However, research of Cartei *et al*.^59^ shows that children adapt their F0 and VTL values when asked to imitate a speaker from the opposite sex, although these adaptations only led to relatively small acoustic changes. These findings seem to indicate that children are already aware of the contributions of F0 and VTL to the perceived gender of adult voices. Also, participants’ JNDs and cue weights were only moderately and weakly correlated for F0 and VTL after correcting for a general effect of age, and only for 4- to 6-year-old children when looking at individual age groups. However, it is difficult to interpret the causality of these results. Categorization of voice cues may be driven by the ability to discriminate these voice cues, but both abilities also may develop independently and improve merely as a function of age. As we only found a significant correlation among 4- to 6-year-old children, of which some showed general low performance on both tasks, children’s different weighting of F0 and VTL seems to be caused primarily by their less robust representations of voice gender categories or task performance rather than their lower discrimination abilities.

## General discussion

The goal of the present study was to investigate the development of children’s ability to discriminate voice gender cues and their weighting of F0 and VTL cues for voice gender categorization. Our results show that there are dissociations in the development of children’s discrimination of F0 and VTL and weighting of F0 and VTL for voice gender categorization. In our first experiment, we found that children’s discrimination of VTL becomes adult-like earlier than their discrimination of F0. In contrast, the results of our second experiment showed that children’s weighting for voice gender categorization becomes adult-like earlier for F0 than VTL. Hence, for both discrimination and cue weighting for voice gender categorization, we found differences in the development of F0 and VTL. However, the dissociation between the development of children’s discrimination of F0 and VTL cues did not correspond to the developmental pattern for F0 and VTL that we found for children’s weighting for voice gender categorization. Finally, we only found a moderate and a small overall significant correlation between children’s F0 and VTL JNDs, i.e., discrimination thresholds, and cue weights after correcting for a general effect of age and merely a significant correlation for 4- to 6-year-old children when looking at individual age groups.

The discrepancy between the developmental patterns for discrimination and categorization of voice gender cues demonstrates that these abilities may rely on different auditory processes. Evidence for the latter theory is provided by Mirman *et al*.^50^, who reported a similar trade-off effect between the discrimination versus categorization based on the acoustic dynamics of voice cues. Dynamic acoustic cues may be more perceptually salient than static cues, which makes listeners rely on them more for categorization. However, due to their rapidly-changing and dynamic nature, differences in dynamic cues may be more difficult to perceive for listeners than static cues. In addition, children’s discrimination of VTL may become adult-like earlier because it has a linguistically more prominent role, as it is essential for the correct interpretation of vowels^30^ while F0 gives more subtle paralinguistic information in non-tonal languages.

Another factor that may have played a role is that children’s representations of voice gender are based on voice gender distinctions among children rather than adults. Fleming *et al*.^60^ found that adult listeners are better at distinguishing speakers’ voices in their native language than in a different language because of a higher familiarity with the phonology of their native language. This finding demonstrates that even adults show a bias in voice perception and discrimination based on auditory experience. Voice gender in children is primarily determined by differences in formant frequencies, possibly caused by differences in the resonator width and size^38^, as there are no clear sex differences in children’s F0 and VTL voice parameters before puberty^37,38^. Hence, children may have different categories of voice gender than adults. Future research should investigate whether adults show similar weighting of F0 and VTL as children for categorizing the voice gender of children’s voices instead of adults’ voices.

Investigating the relationship between different tasks and their underlying cognitive processes is also eminent for research on other perceptual learning mechanisms, as similar differences in developmental patterns and parameters may apply. For instance, Leibold *et al*.^61^ found that children showed a similar or larger benefit from voice gender differences between speakers for speech perception in competing speech than adults, while Flaherty *et al*.^13^ showed children did not benefit from differences in only speakers’ F0. A potential explanation for this discrepancy given by the authors was that children may rely more on combined differences in F0 and VTL cues to segregate speakers based on voice gender. However, our results from Experiment 2 suggest that children use and rely on differences in both F0 and VTL for categorizing speakers’ voice gender when the differences are sufficiently large. Understanding these developmental patterns is also particularly relevant for research with clinical populations, such as children with hearing impairments^45,62^, as their difficulties in perceiving and recognizing voices may originate from different sources, such as perceptual limitations or difficulty of allocating attention.

In conclusion, we found that children’s discrimination and weighting for voice gender categorization develop differently for F0 and VTL. Furthermore, both abilities show different developmental patterns for F0 and VTL, implying that these abilities inherently rely on different auditory processes and differ based on the nature of the acoustic cue. Generally, we also found that children’s abilities to discriminate and weigh voice cues for categorization take a long time to mature, which corresponds with the earlier reported findings on the discrimination and categorization of phonemes^17,18^. In addition, our findings emphasize that voice perception research should take into account how the development of various perceptual abilities, for instance, voice discrimination, categorization, and identification, are related and on which parameters they rely.

## Data Availability

The datasets generated and analyzed during the current study are available in the Dataverse NL repository, https://hdl.handle.net/10411/2MSHYR.
